# pH and Redox Dual-Responsive MSN-S-S-CS as a Drug Delivery System in Cancer Therapy

**DOI:** 10.3390/ma13061279

**Published:** 2020-03-12

**Authors:** Yanqin Xu, Liyue Xiao, Yating Chang, Yuan Cao, Changguo Chen, Dan Wang

**Affiliations:** School of Chemistry and Chemical Engineering, Chongqing University, Chongqing 401331, China; xuyanqin666@163.com (Y.X.); XiaoLiyue777@163.com (L.X.); 13271242585@163.com (Y.C.)

**Keywords:** mesoporous silica nanoparticles, double response, drug delivery, drug release

## Abstract

In order to achieve a controlled release drug delivery system (DDS) for cancer therapy, a pH and redox dual-responsive mesoporous silica nanoparticles (MSN)-sulfur (S)-S- chitosan (CS) DDS was prepared via an amide reaction of dithiodipropionic acid with amino groups on the surface of MSN and amino groups on the surface of CS. Using salicylic acid (SA) as a model drug, SA@MSN-S-S-CS was prepared by an impregnation method. Subsequently, the stability, swelling properties and drug release properties of the DDS were studied by x-ray diffraction, scanning electron microscopy, Fourier transform infrared microspectroscopy, size and zeta potential as well as Brunauer–Emmett–Teller surface area. Pore size and volume of the composites decreased after drug loading but maintained a stable structure. The calculated drug loading rate and encapsulation efficiency were 8.17% and 55.64%, respectively. The in vitro drug release rate was 21.54% in response to glutathione, and the release rate showed a marked increase as the pH decreased. Overall, double response functions of MSN-S-S-CS had unique advantages in controlled drug delivery, and may be a new clinical application of DDS in cancer therapy.

## 1. Introduction

Recently, various chemotherapeutics have been exploited for effective cancer treatments. However, traditional therapeutic drugs show significant adverse effects on healthy organs. Nano-drugs have controlled release or targeting, which may alleviate the serious side effects of cytotoxic chemotherapeutic drugs to a certain extent [1,2,3,4]. Mesoporous materials have an ordered pore structure and a high specific surface area, which can provide a structure capable of accommodating a large number of drugs [5]. On the other hand, an ordered pore network can appropriately control the loading and release of drugs. These advantages have attracted attention in the field of application of drug delivery systems (DDS) [6,7]. Among various mesoporous materials [8,9,10], the unique mesopore structures [11] and facile surface functional properties [12,13] of mesoporous silica nanoparticles (MSN) make it feasible for use in the design of diverse stimuli-responsive gatekeepers. These structures control the opening and closing of the mesopores for a triggered release of drugs loaded inside the porous particles [14,15]. So far, several materials, such as inorganic nanoparticles [16,17], dendrimers [18,19], rotaxane [20], cyclodextrin [21] and DNA [22] have been applied to cap the mesoporous openings on the surface of MSN. These materials permit different external triggering motifs such as redox state changes [23], pH [24], temperature [25], illumination [26], enzyme activity [27] and so on to achieve a controlled release of guest drugs from the pores. However, the side effects of these cancer therapeutics cannot be ignored in clinical practice [28]. Therefore, there is an urgent need to develop effective stimuli-responsive nanocarriers to improve targeted drug release.

The amino group in chitosan (CS) can be protonated at a certain pH range, revealing a possible pathway for using CS as a smart molecular device. Wu et al. [29] successfully prepared a pH-responsive CS hydrogel film and attached it to a porous silica layer. The successful synthesis of CS as a pH-responsive nanoparticle valve that controls the release of insulin confirmed that CS can be used as a pH-sensitive composite. Using this as a foundation, we synthesized a drug delivery system MSN-S-S-CS based on CS-terminated MSN nanoparticles to facilitate glutathione (GSH) triggered release.

The surface of MSN was modified with a disulfide compound and the mesopores of MSN were covalently capped by CS through a disulfide linkage to prevent drug leakage during circulation. Due to the acidity of the microenvironment of cancerous tissue [30], the -NH_2_ molecule can be protonated and subsequently, the CS polymer chain gradually dissolves, releasing drug molecules [31,32]. As the concentration of GSH in the intracellular matrix of cells is 10^2^–10^3^ times higher than that in the extracellular environment [33], the disulfide bond would break in response to GSH. Following that, the CS gatekeeper is removed from the surface of MSN, triggering the release of the drug once the nanocarriers were internalized into tumor cells.

Our MSN-SS-CS drug delivery system was designed according to microenvironment differences between tumor and normal tissue cells [34], combined with the pH sensitivity of CS, and the redox responsiveness of disulfide bonds. These characteristics significantly enhanced the cancer cell targeting capabilities of DDS, greatly reducing the chance of internalization by normal cells during drug delivery [35]. In our protocol, we studied the stability, swelling performance and drug release of MSN-SS-CS. Our experimental results demonstrate that the MSN-SS-CS has the duality of pH and redox response. When the two conditions coexisted, the response was synergistic and the release rate was higher than it was under a single condition. Considering these advantages, the MSN-SS-CS is a promising drug controlled release material for use in tumor therapeutics.

## 2. Materials and Methods

### 2.1. Materials

Tetraethoxysilane (TEOS), 3-aminopropyltriethoxysilane (APTES), cetyl trimethyl ammonium bromide (CTAB), N-hydroxysuccinimide (NHS), 3-dithiodipropionic acid, phosphate buffer solution (PBS), 1-ethyl-3-(3-dimethylaminopropyl)carbodiimide (EDC), salicylic acid (SA), chitosan (CS), glutathione (GSH) and all other chemicals were purchased from Aladdin (Shanghai, China), unless otherwise noted. All other chemicals were reagent grade and used without further purification.

### 2.2. Preparation of MSN and MSN-S-S-CS 

Mesoporous silica nanoparticles were synthesized by the template method, using CTAB as the template and TEOS as the silicon source. To form a self-supporting skeleton structure, TEOS was polymerized on the surface of CTAB. An amount of CTAB (0.3 g) was dissolved into a mixture of 30 mL double distilled water and 10 mL anhydrous ethanol. Afterwards, an NH_3_ H_2_O solution was added into the above solution under stirring to adjust the pH to between 9 and 10. One point two milliliters of TEOS was added dropwise into the solution under vigorous stirring until a homogeneous solution was obtained. The resulting white precipitate remained static for 5 h, was separated by centrifugation, washed three times with the distilled water and absolute ethanol mixture, and dried at 60 °C for 12 h. The template CTAB was removed by calcination in a muffle furnace at 550 °C for 5 h to obtain pore-ordered MSN.

Two hundred milligrams of MSN were uniformly dispersed in 20 mL of deionized water for 10 min. After the addition of acetic acid and APTES, the mixture was stirred for 24 h, centrifuged, washed and dried to obtain aminated MSN-NH_2_. The MSN-NH_2_ was dissolved in PBS, and 3,3-dithiodipropionic acid, EDC and NHS were added sequentially. The mixed solution was stirred for 12 h, centrifuged, washed, and dried in vacuo to prepare the MSN-S-S-COOH for the next step. Finally, the MSN-S-S-COOH was dissolved in PBS at pH 5.8. The same procedure was used to prepare MSN-S-S-CS, except CS was added.

### 2.3. Characterization

To investigate the microstructure of the composite, X-ray diffraction (XRD, XRD-6000 Shimadzu Corporation, Kyoto, Japan) patterns were acquired with Cu-Ka radiation in the 2θ range of 2°–5°, and Fourier transform infrared (FTIR, MagnaIR550II Nicolet, Waltham, MA, USA) spectroscopic analysis was carried out via the standard KBr disk method in the wavelength range of 400–4000 cm^−1^. We also analyzed the morphology and microstructure of the composites via scanning electron microscopy (SEM, JSM-6490LV FEI), Brunauer–Emmett–Teller surface area (BET, ASAP2020M Micromeritics, Norcross, GA, USA) and the laser particle size analyzer (Zetasizer Nano Malvern, Malvern, UK).

### 2.4. Determination of Stability and Responsiveness 

Because SA is a hydrophobic drug, ethanol is used as a solvent. Therefore, the stability of MSN and MSN-SS-CS must be studied under ethanol conditions, and under different pH and GSH conditions. We measured the changes in particle size and zeta potential under ethanol as well as any changes in particle size under simulation of normal and diseased tissue fluids.

### 2.5. Drug Loading and Body Fluid Simulation

Two hundred milligrams MSN-S-S-CS was mixed with SA in absolute ethyl alcohol (2 mg/L). After stirring for 24 h, SA@MSN-S-S-CS was centrifuged and washed. These samples were dried under vacuum drying, sealed, and stored until analysis. To determine drug loading content, the absorbance of the drug in residual liquid was measured by recording the absorption spectra at 293 nm using a UV–VIS spectrophotometer (Cintra-10e, Australia GBC), and the calibration curve was obtained with SA solutions at different concentrations. The drug loading capacity (DLC) and drug loading efficiency (DLE) were calculated by the following formulas:

DLC = 100% × (weight of loaded SA)/(weight of MSN-S-S-CS)

DLE = 100% × (weight of loaded SA)/(weight of SA in feed)

The simulation was performed under the following conditions:

(1) Simulated gastric juice (pH 1.2): 2.0 g NaCl, 3.2 g pepsin and 7 mL hydrochloric acid, distilled water added to dissolve to a volume of 1000 mL;
(2) Simulated intestinal fluid (pH 7.4): 0.3 g KH_2_PO_4_, 2.88 g Na_2_HPO_4_•7H_2_O, 0.20 g KCl, 8 g NaCl, dissolved in distilled water, and diluted to 1000 mL;
(3) Simulated large intestinal fluid (pH 8.4): 3.5 g Na_2_HPO_4_•7H_2_O, 3.5 g KH_2_PO_4_, diluted to 1000 mL in distilled water, and adjusted to pH to 8.3 with dilute sodium hydroxide solution;
(4) PBS buffer solution (pH 5.8): 8.3 g KH_2_PO_4_ and 0.9 g K_2_HPO_4_, dissolved in distilled water, diluted to 1000 mL;
(5) 2 mM GSH solution: 0.6 g GSH dissolved in distilled water, and volume adjusted to a 1000 mL volumetric flask and shaken prior to use;
(6) 10 mM GSH solution: dissolved 3.0 g GSH in distilled water in a 1000 mL volumetric flask, and shaken while standing;
(7) pH 1.2 + 10 mM GSH, pH 5.8 + 10 mM GSH, pH 7.4 + 10 mM GSH: three aliquots of 3.0 g GSH were dissolved in the above 1000 mL pH solutions.

### 2.6. In Vitro Release

Nine parts of 200 mg SA@MSN-S-S-CS were weighed and placed in an Erlenmeyer flask mixed with the solution prepared in the previous step. The Erlenmeyer flask was placed in a shaker at 37°C with an oscillation rate of 50 rpm. Three milliliters of the mixed solution was taken at regular intervals and 3 mL of the original buffer solution was added. The absorbance was measured at 293 nm by UV–VIS spectrophotometer (Cintra-10e GBC, Australia), and was measured in parallel three times. The cumulative release rate (CR) was calculated according to the standard curve method as follows:

m_n_ = (C_1_ + C_2_ + C_n-1_) × 0.03 + C_n_ × 0.1

CR = 100% × m_n_/m_0_

Cn is the mass concentration (mg/L) of SA in the nth sampling, m_n_ is the cumulative release mass (mg) of the nth sampling SA, and m_0_ is the total mass (mg) of the loaded drug. 

Drug release curves were drawn according to CR.

## 3. Results and Discussion

### 3.1. Synthesis and SEM Characterization of MSN and MSN-S-S-CS

**Figure 1:**
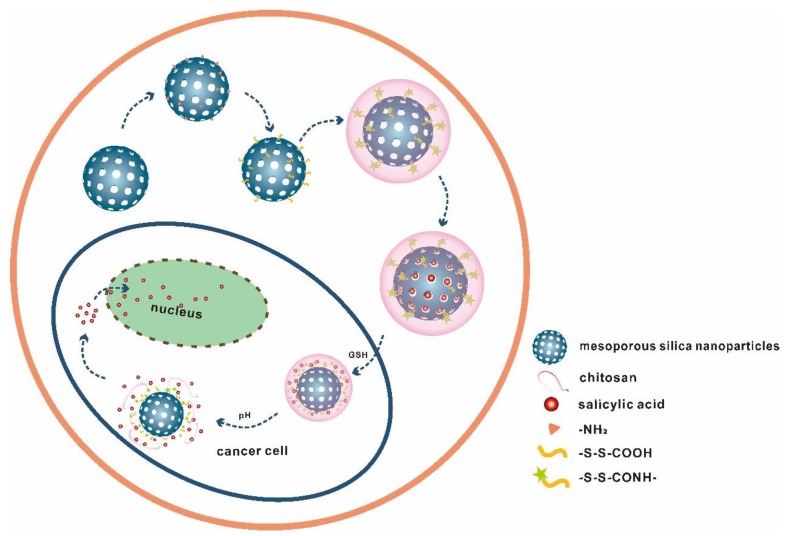
Schematic of the synthesis and responsive release of pH/redox double-reactive MSN-S-S-CS.

**Figure 2:**
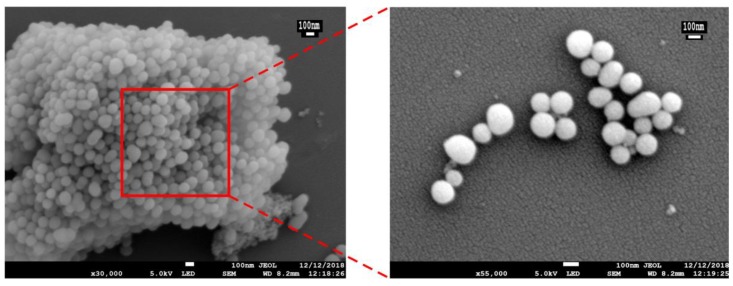
SEM images of mesoporous silica nanoparticles (MSN).

The preparation and response release principle of MSN-S-S-CS is shown in Figure 1. Firstly, TEOS was polymerized on the surface of CTAB to form a self-supporting skeleton structure. Subsequently, CTAB was removed by high-temperature calcination to obtain pore-ordered MSN with smooth spherical structures with a diameter of about 100 nm (Figure 2). A highly ordered mesoporous structure [36] with an MSN array is shown in the corresponding high-powered SEM image. After reaction with APTES, the surface of the MSN was grafted with amino groups to produce aminated MSN-NH_2_. Finally, 3,3-dithiodipropionic acid was reacted with amino groups grafted on the surface of mesoporous silica to form MSN-S-S-COOH, which was subsequently reacted with an amino group on CS to obtain MSN-S-S-CS.

The SA@MSN-S-S-CS (obtained by impregnation [37] loading SA) entered the MSN channel by diffusion, and the drug-loaded molecules entered the simulated diseased environment. Due to the higher concentration of GSH and lower pH, the -NH_2_ in CS gradually protonated to -NH_3_^+^, the water solubility was enhanced, and the water molecules entered the CS molecular chain. In addition, GSH underwent a redox reaction, breaking the disulfide bonds [38]. As a result, the valve wrapped on the surface of the MSN pore was directly opened, and the drug molecule released. However, in a normal tissue environment, the response degree was smaller due to the lower concentration of GSH as compared to diseased tissue. 

### 3.2. Brunauer–Emmett–Teller Surface Area (BET) Analysis

**Figure 3:**
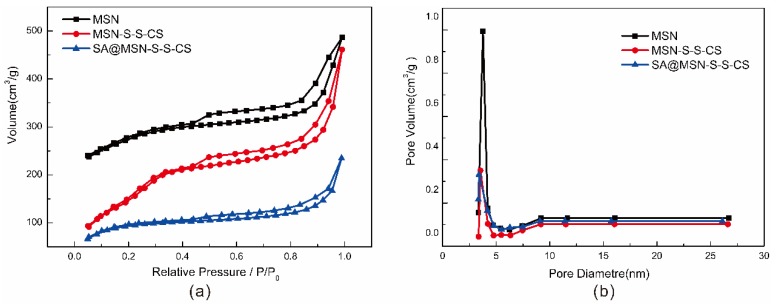
N_2_ adsorption-desorption curve (**a**) and diameter distribution (**b**) of MSN, MSN-S-S-CS and SA@MSN-S-S-CS.

The pore characteristics of MSN, MSN-S-S-CS and SA@MSN-S-S-CS were analyzed by measuring the nitrogen adsorption-desorption isotherm and BET. The isothermal curves (Figure 3) of MSN-S-S-CS belonged to the type IV adsorption isotherm. Clearly, the condensation step for mesopores was between the relative pressures of 0.2 and 0.3. This indicated very small mesopores, and hysteresis loops were absent in this range. For MSN, the isotherm more closely resembled type I (microporous materials). For SA@MSN-S-S-CS, the isothermal curves were more similar to type II (macroporous solids), indicating that all internal particle pores were blocked. For all isotherms, only type H4 hysteresis was seen. This hysteresis loop resulted from platelet particle assemblies, or loosely aggregated particles forming slit-shaped pores (pores between particles) [39]. The specific surface area of MSN was 1080.22 m^2^/g, the pore diameter was 3.769 nm, and the pore volume was 0.453 cm^3^/g (Table 1). After grafting the amino groups, 3,3-dithiodipropionic acid and CS, the specific surface area was sharply reduced to 654.01 m^2^/g as the majority of CS was grafted onto the surface. In addition, a fraction of the amino molecules entered the mesoporous channel and occupied a part of the tunnels, so that the pore volume and the pore diameter were also reduced. The CS polymer covered the mesoporous structure and prevented drug leakage. After drug loading, the specific surface area of the material decreased to 356.3 m^2^/g, the pore size and pore volume decreased to 3.516 nm and 0.233 cm^3^/g, respectively, indicating that the drug was loaded to MSN in the channel. Here, the BJH pore size distributions were calculated using the desorption branch of the isotherms. The maximum in each curve resulted from the point where the H4 hysteresis closed. Consequently, all samples had the same pore widths.

### 3.3. XRD Analysis

**Figure 4:**
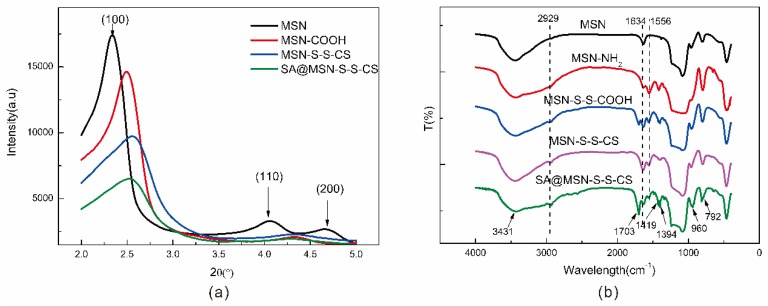
XRD diffraction pattern of MSN and its composites (**a**); FTIR spectra of MSN, MSN-NH_2_, MSN-S-S-CS and SA@MSN-S-S-CS (**b**).

As noted from the XRD diffraction pattern (Figure 4a), MSN showed a strong, sharp diffraction peak and two weaker diffraction peaks. There was a strong peak (100) at 2θ = 2.3°, and diffraction peaks (110, 200) appeared at 2θ = 4°~5°, which were consistent with the literature [40]. The ordered hole structure was a flat hexagonal structure with symmetry of p6mm. *d*_100_ spacing (calculated from powder X-ray diffraction patterns), unit cell parameter (a_0_ = 2*d*_100_/√3), and the value of a_0_ is shown in Table 2. The diffraction peaks of the three crystal faces were consistent with the characteristic diffraction peaks of MCM-41 mesoporous silica, indicating that the synthesized MSN had a long-range ordered hexagonal-phase pore structure with diffraction peaks modified on the surface [41]. With the modification of its surface, the intensity of the diffraction peak was gradually weakened, the peak position shifted, and the degree of order was reduced. This also indicated that some of the reactants entered the mesoporous channel during the modification process. Following drug loading, most of the drug molecules entered the mesoporous channels of MSN-SS-CS, resulting in the diffraction peak broadening and the intensity weakening.

### 3.4. FTIR Analysis

The drug loading of MSN was examined by FTIR spectroscopy. Vibration peaks, such as 3431 cm^−1^, 792 cm^−1^, 462 cm^−1^, and 960 cm^−1^, were characteristic absorption peaks typical of MSN (Figure 4b) [42]. At 1556 cm^−1^, there was an asymmetric bending vibration of the N-H bond, which is a characteristic peak of the amino group. At 1426 cm^−1^, there was a stretching vibration peak of the C-N bond, indicating that the amino group was successfully grafted onto the surface of the MSN. After the modification of 3,3-dithiodipropionic acid, a vibrational peak of the carboxyl group appeared at 1692 cm^−1^, and a small peak appeared at 2929 cm^−1^, which was the stretching vibration peak of the ethyl group in 3,3-dithiodipropionic acid. After CS modification, the carboxyl peak disappeared and there was a distinct peak of the amino group of CS at 1556 cm^−1^, indicating that CS was attached to the surface. After the drug was loaded, a vibrational peak of the carboxyl group at 1703 cm^−1^, and vibration peaks of the benzene ring at 1419 cm^−1^ and 1394 cm^−1^ appeared, a characteristic peak of SA.

### 3.5. Size and Zeta Potential

**Figure 5:**
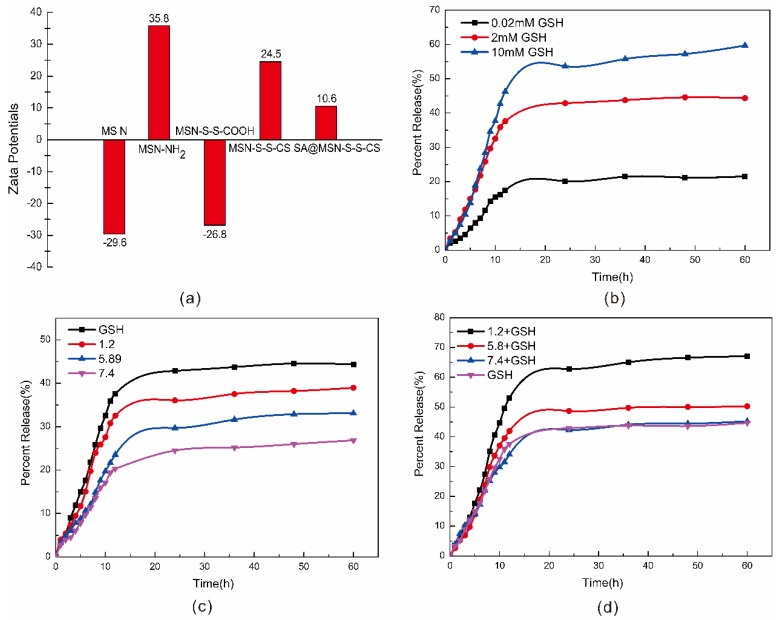
Zeta potential measurements of MSN, MSN-NH_2_, MSN-S-S-COOH, MSN-S-S-CS and SA@MSN-S-S-CS (**a**); Cumulative drug release curves at different concentrations of glutathione (GSH) (**b**) and different pH (**c**); Release profile of SA@MSN-S-S-CS in 2 mM GSH solution at different pH (**d**).

The laser particle size analyzer showed that the surface of the MSN was rich in hydroxyl groups, such that MSN had a lower potential surface with a negative charge [43] and a potential of −29.6 mV (Figure 5a). Due to the protonation of the amino group, when the amino group was grafted, the potential increased to 35.8 mV. After grafting the carboxyl group and CS, the potential notably changed to −26.8 mV and 24.5 mV, respectively. The decarboxylation of the carboxyl group exhibited a negatively charged -COO-. After grafting CS, the amino group played a significant role. After the drug was loaded, SA had both hydroxyl and carboxyl groups, reducing the overall drug loading material needed. 

### 3.6. Stability and Responsiveness Analysis of MSN-S-S-CS

The particle size change and zeta potential of MSN and MSN-S-S-CS were determined by using ethanol as a solvent (Table 3). MSN was essentially stable in ethanol solution. Over time, the particle size changed marginally and the polymer dispersity index (PDI) was <0.3, indicating that the particle size dispersion was relatively uniform [44]. However, the particle size of MSN-SS-CS in ethanol had some irregularity, possibly due to some unstable factors in the measurement process, such as partial agglomeration. 

**Figure 6:**
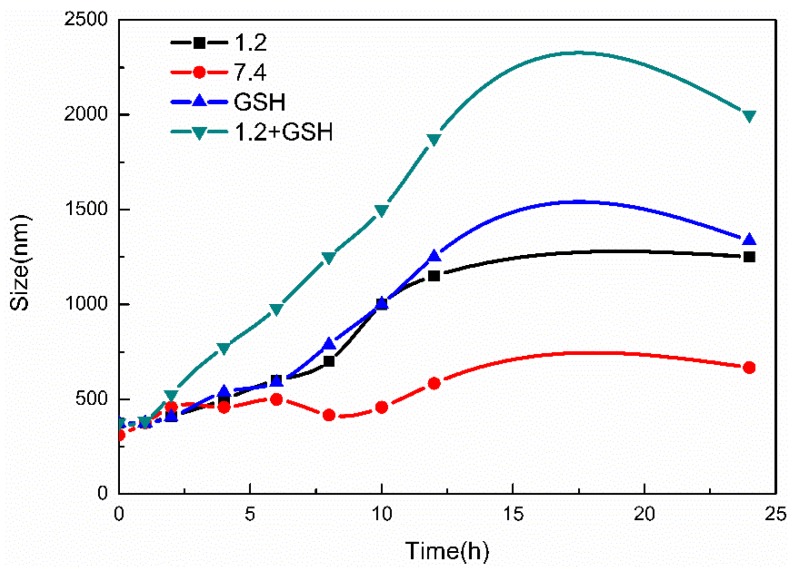
Particle size changes of MSN-S-S-CS in different buffer solutions.

Under different pH conditions and in the presence of GSH, the MSN-S-S-CS particle size exhibited a considerable change (Figure 6). Under strong acidic conditions, the -NH_2_ molecule was completely protonated and water molecules were able to enter the internal structure [32]. The particle size of MSN-S-S-CS increased as the pH decreased, for example, in GSH solution, the particle size increased from 386 nm to 1489 nm. In contrast, in GSH solution with pH 1.2, the particle size changed from 368 nm to 2008 nm. On the one hand, this change could be due to the protonation of CS [45]. On the other hand, we found that the effect of GSH on the particle size was greater than the effect of pH, indicating that the disulfide bond can be broken in response to GSH [46]. However, when the particle size was measured by the particle size analyzer, we found that the PDI constant was large and the particle dispersion was uneven. It is possible that CS was detached from MSN after the disulfide bond was broken. Overall, MSN-S-S-CS had good pH and GSH responsiveness.

### 3.7. Simulation Experiment

Using the experimental data, the drug loading rate was calculated to be 8.17%, and the entrapment rate was 55.64%. Figure 5 shows the cumulative drug release profile of the drug for pH and GSH concentration. As shown in Figure 5b, as the GSH concentration increased, the drug release rate increased. At GSH concentrations of 0.02 mM, 2 mM, and 10 mM, the drug release rates were 21.54%, 44.35%, and 59.70%, respectively. At higher concentrations of GSH, the disulfide bond was cleaved to release the drug, clearly demonstrating that MSN-S-S-CS has good redox responsiveness. As the pH increased, the drug release rate decreased, and the composite also had pH responsiveness (Figure 5c). Moreover, when the concentration of GSH was 2 mM, the release rate was higher than it was under different pH conditions. The redox response of MSN-S-S-CS was greater than the pH response. Figure 5d shows the cumulative release profile at the coexistence of different pH levels and 2 mM GSH. For example, with physiological pH 7.4 and GSH together in buffer solution, the release rate was 45.23%, which was similar to that of the GSH solution alone. Under the condition of pH 5.8 and GSH coexistence, the drug release rate was 50.21%, which was higher than the drug release rate under single conditions. At pH 1.2 and GSH coexistence, the release rate was as high as 67.08%, which was also higher than the release rate at pH 1.2 and 2 mM GSH alone. The above circumstances demonstrated the synergy of the composite with pH and redox response. 

## 4. Conclusions

In our experiment, the natural macromolecular compound CS with good biocompatibility was used as the blocking molecule of the drug. The MSN-SS-CS material was synthesized by the amide reaction of the dithiodipropionic acid with the amino group on the surface of MSN and the amino group on the surface of CS. Using SA as a model drug, the drug-loading system was characterized by XRD, FTIR, BET and zeta potential. The in vitro drug release experiment was performed with a simulation of human body fluid to study the effects of pH and GSH on drug release.

The characterization analysis showed that the MSN-SS-CS composite was successfully synthesized, and the drug was successfully loaded and encapsulated. The determination of the particle size of MSN and MSN-SS-CS in ethanol solution showed that the particle size of MSN and MSN-SS-CS changed little over time, indicating that the synthesized material had a stable structure and was suitable for hydrophobic drugs. The drug release test proved that SA@MSN-S-S-CS has pH and GSH dual responsiveness. At the same time, the drug response rate controlled by the pH response was less than the drug release rate controlled by the redox response. Therefore, the degree of redox responsiveness was greater than the degree of pH response. When the two conditions coexisted, the release rate was higher than it was under the single condition. Our results show that the MSN-SS-CS composite had pH and redox responsiveness, which together had a synergistic effect.

## Figures and Tables

**Table 1:** Brunauer–Emmett–Teller surface area (BET) structure parameters of MSN-NH_2_, MSN-S-S-CS and SA@MSN-S-S-CS.

No.	Specific Surface Area (m^2^/g)	Aperture (nm)	Pore Volume (cm^3^/g)
MSN	1080.22	3.769	0.453
MSN-S-S-CS	654.01	3.746	0.416
SA@MSN-S-S-CS	356.30	3.516	0.233

**Table 2:** Crystallographic parameters of the MSN and its composites.

No.	2θ (°)	*d*_100_ (nm)	a_0_ (nm)
MSN	2.34	3.764	4.272
MSN-COOH	2.50	3.533	4.108
MSN-S-S-CS	2.56	3.449	4.010
SA@MSN-S-S-CS	2.52	3.499	4.068

**Table 3:** Changes in particle size and potential of MSN and MSN-S-S-CS in C_2_H_5_OH.

Time (h)	SN		MSN-S-S-CS	
	PDI	ζ (mV)	PDI	ζ (mV)
0	0.267	−20.3	0.432	7.8
1	0.331	−26.7	0.608	5.7
2	0.275	−24.6	0.54	4.6
4	0.432	−19.8	0.396	7.4
6	0.341	−21.6	0.324	8.8
8	0.473	−16.8	0.732	5.9
10	0.212	−23.9	0.473	7.5
12	0.435	−17.4	0.401	8.6
